# Arachidonic acid increases matrix metalloproteinase 9 secretion and expression in human monocytic MonoMac 6 cells

**DOI:** 10.1186/1476-511X-8-11

**Published:** 2009-03-30

**Authors:** Tiina Solakivi, Tarja Kunnas, Satu Kärkkäinen, Olli Jaakkola, Seppo T Nikkari

**Affiliations:** 1 Department of Medical Biochemistry, University of Tampere Medical School, Tampere, Finland; 2 Institute of Medical Technology, University of Tampere, Tampere, Finland; 3 Department of Clinical Chemistry, Tampere University Hospital, Tampere, Finland

## Abstract

**Background:**

Dietary fatty acids may modulate inflammation in macrophages of the atherosclerotic plaque, affecting its stability. The n-6 polyunsaturated fatty acid (PUFA) arachidonic acid (AA) generally promotes inflammation, while the PUFAs of the n-3 series eicosapentaenoic acid (EPA), docosapentaenoic acid (DPA) and docosahexaenoic acid (DHA) are considered anti-inflammatory. We determined how these PUFAs influence MMP-9 expression and secretion by the human monocytic cell line (MonoMac 6) at baseline and after 24-hour exposure. MMP-9 protein was measured by zymography and relative levels of MMP-9 mRNA were determined using quantitative real time PCR.

**Results:**

Supplementation with AA (but not the n-3 fatty acids) increased, in a dose-dependent manner, expression of MMP-9 protein. This stimulation was regulated at the mRNA level. MMP-9 secretion started after 1 h of incubation and could not be prevented by simultaneous presence of n-3 series fatty acids. Finally, the secretion could be attenuated by LY 294002, a specific phosphatidylinositol-3-kinase (PI3K) inhibitor and by SH-5, a selective Akt inhibitor, suggesting that activation of PI3K by AA leads to augmented and sustained MMP-9 production.

**Conclusion:**

This study shows that of the PUFA studied, AA alone influences the expression of MMP-9, which might have implications in MMP-9 induced plaque rupture.

## Background

Dietary fatty acids are known to modulate the metabolism of lipids and lipoproteins and therefore also to be involved in cardiovascular and metabolic diseases [1,2]. Dietary polyunsaturated fatty acids (PUFA) are classified into two families, the n-6 and n-3 series. Although both families are substrates for the same enzymes in many cellular processes, the affinity of enzymes is greater for the n-3 family than that for the n-6 family. As an example, arachidonic acid (20:4n-6, AA), a metabolite of linoleic acid (18:2n-6), is the substrate of cyclooxygenases and lipoxygenases in the production of potent inflammatory eicosanoids. The polyunsaturated fatty acids of the n-3 series (eicosapentaenoic acid 20:5n-3, EPA; docosapentaenoic acid 22:5n-3, DPA; and docosahexaenoic acid 22:6n-3, DHA) limit the synthesis of these mediators from AA, and enhance the synthesis of less inflammatory eicosanoids from EPA [3]. Recent studies have identified novel groups of powerful EPA and DHA derived anti-inflammatory mediators which are produced during the resolving phase of an acute inflammatory response [4].

The propensity of atherosclerotic plaques to rupture is influenced by their lipid content and the distribution of lipid within the plaque as well as by the extent of infiltration of macrophages at the shoulder regions of the plaque and by the thickness of the fibrous cap [5]. A thin fibrous cap and a substantial presence of macrophages and other inflammatory cells in the shoulder region indicate a vulnerable plaque likely to rupture. Matrix metalloproteinases (MMPs) are a group of enzymes whose major functions are directed toward remodelling of extracellular matrix (ECM) components. Gelatinase B (MMP-9) is a very complex enzyme in terms of domain structure or regulation of its activity and expression. MMP-9 activity is controlled at different levels: transcriptional activation of the gene by cytokines and other factors, activation of the pro-enzyme by various enzymes like serine proteases and regulation by specific tissue inhibitors of the matrix metalloproteinases (TIMPs). MMP-9 has been shown to be up-regulated in unstable angina pectoris. It is mainly expressed by macrophages located especially in vulnerable regions of the atherosclerotic plaque [6]. Published results thus suggest that localized increase in MMP-9 associated with an inflammatory process in the vascular wall has the potential to weaken the tissue structure thus increasing the risk for plaque rupture. Still, the signalling pathways that lead to induction of expression of MMP-9 are incompletely understood.

Dietary n-3 fatty acids have a variety of anti-inflammatory and immuno-modulating effects that may be of relevance to atherosclerosis and its clinical manifestations of myocardial infarction, sudden death and stroke [1]. There is epidemiological evidence that consumption of fish or n-3 PUFA protects against cardiovascular disease [7]. However, more critical meta-analyses regarding the clinical importance of n-3 PUFA have also demonstrated no clear effect on combined cardiovascular events [8]. The effects of n-3 PUFA have been variously ascribed to anti-arrhythmic or anti-thrombotic actions of n-3 PUFA but might as well be linked to their anti-inflammatory effects [1]. Some of these effects are brought about through modifications in gene expression since long chain fatty acids also serve as ligands for several nuclear transcription factors [9]. In a recent study, patients who were scheduled to carotid endarterectomy were randomly allocated to receive either placebo, sunflower oil or fish oil. It was shown that the n-3 PUFAs were readily incorporated into plaques and were associated with reduced numbers of macrophages and signs of inflammation [10]. Inflammatory prostaglandins and leukotrienes derived from arachidonic acid have previously been shown to induce MMP production [11-13]. Human MonoMac 6 monocytes appear to be a useful cellular model to investigate effects of compounds on plaque vulnerability through MMP-9 activity [14]. In this study we sought to clarify relationships between arachidonic acid and the polyunsaturated n-3 fatty acids in regulation of MMP-9.

## Results

**Figure 1:**
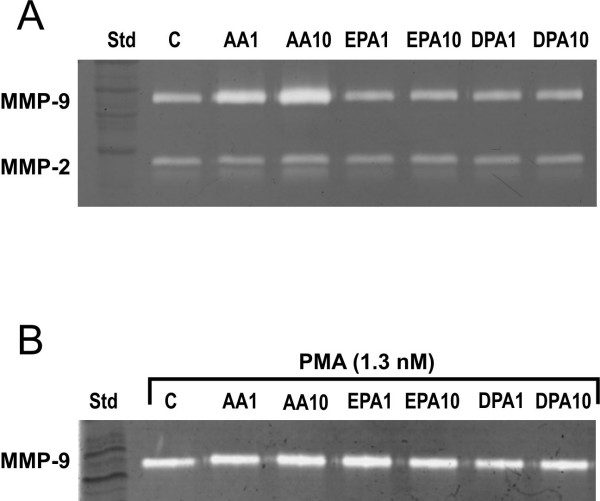
**Effect of polyunsaturated fatty acids and PMA on the production of MMP-9**. MonoMac 6 cells (0.8 × 10^6^/ml in serum-free medium X-Vivo 15) were stimulated with 1 or 10 μM arachidonic acid (AA), eicosapentaenoic acid (EPA) or docosapentaenoic acid (DPA) without (1A) or in the presence of 1.3 nM PMA (1B). The culture media were harvested after 24 h incubation and analysed for MMP-9 by gelatin zymography. The media of PMA-treated cells were diluted (1:10) to preserve the linearity of response in image analysis.

To investigate the impact of long chain fatty acids with different characteristics on MMP-9 secretion we incubated MonoMac 6 cells in the presence of 1 μM and 10 μM AA, EPA or DPA (Fig. 1A). The secretion of MMP-9 to the medium increased in the presence of 1 μM AA as shown by the greater activity of MMP-9 in zymography (2.3 ± 0.2-fold, n = 10) and was further enhanced by 10 μM AA (6.7 ± 1.0-fold, n = 14). EPA and DPA had no effect, neither did DHA (result not shown). The effect of PMA (1.3 nM), a known activator of monocytes and also of MMP-9 secretion [15] was about ten-fold greater than that of 10 μM arachidonic acid Addition of 1 μM and 10 μM AA, EPA or DPA along with PMA did not affect MMP-9 production (Fig. 1B).

Fatty acid analysis of the cells showed that all the long chain polyunsaturated fatty acids were readily taken up by the cells and metabolized further. The amount of AA in cellular lipids increased in the presence of 1 μM AA from 1.3 ± 0.037 μg/1.0 × 10^6 ^cells to 2.2 ± 0.23 μg/1.0 × 10^6 ^cells, and in the presence of 10 μM AA to 7.4 ± 1.29 μg/1.0 × 10^6 ^cells. In addition, fatty acid analysis showed that AA was efficiently elongated to 22:4n-6. Similarily, EPA was taken up by the cells and was elongated to 22:5n-3, since the amount of this fatty acid increased from 0.5 ± 0.046 μg/1.0 × 10^6 ^cells to 2.2 ± 0.28 μg/1.0 × 10^6 ^cells when the cells were exposed to 10 μM EPA for 24 h. Furthermore, when the monocytes were incubated in the presence of DPA this fatty acid was metabolized to 22:6n-3, which was further retroconverted to EPA. These results show that the fatty acids were taken up and were available at least to the enzymes in endoplasmic reticulum and peroxisomes.

**Figure 2:**
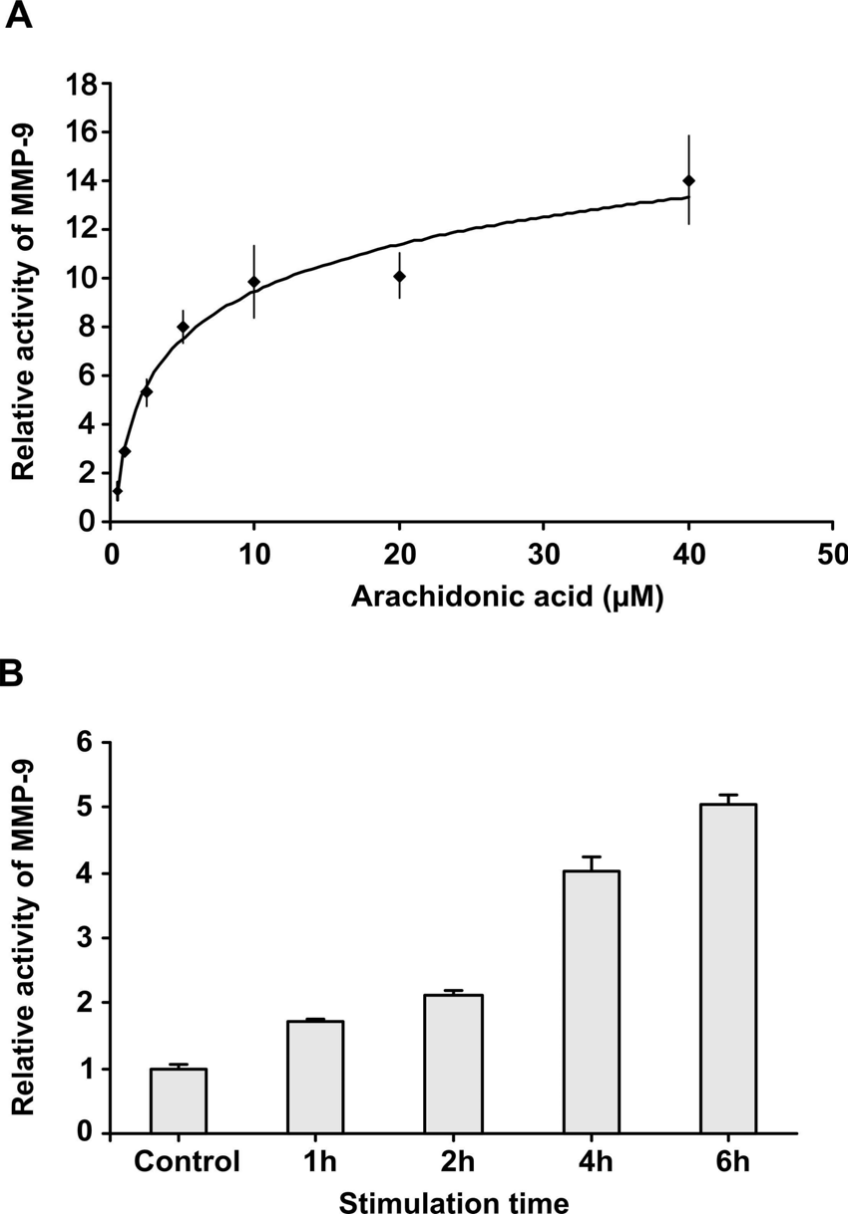
**Dose- and time-dependent stimulation of MMP-9 secretion by arachidonic acid**. **2A**. MonoMac 6 cells (0.8 × 10^6 ^cells/ml in X-Vivo 15) were treated for 24 h with increasing concentrations of arachidonic acid. The media were collected and analysed for MMP-9 by zymography. Data are shown as means ± SEM of 3 to 4 independent experiments. The increase of MMP-9 secretion was statistically significant from 2.5 μM AA and upwards. **2B**. MonoMac 6 cells (0.8 × 10^6 ^cells/ml in X-Vivo 15) were pre-exposed to 10 μM arachidonic acid for 1, 2, 4 or 6 hours where after the culture medium was changed to X-Vivo without arachidonic acid for 24 h. The media were analysed for MMP-9 by zymography. Results are shown as means ± SEM of three independent experiments. A statistically significant increase in MMP-9 secretion was evident after 1 h preincubation in comparison with cells incubated in the presence of vehicle only.

We then looked at the concentration-dependency of arachidonic acid-induced secretion of MMP-9. We incubated MonoMac-6 cells for 24 h in the presence of increasing concentrations of AA (0–40 μM). Analysis of the zymograms by image analysis gave a logarithmic dose response curve (Fig. 2A). Examination of the cells after incubation in the presence of 40 μM AA revealed large clumps of clustered cells. This phenomenon was associated with a slightly lowered cellular protein concentration suggesting some undesirable effects of higher concentrations of AA on cellular well-being.

To investigate the time course of AA-induced MMP-9 secretion we took small samples of medium at 1, 2, 4, 8, 16 and 24 h after adding 10 μM AA or 1.3 nM PMA. MMP-9 activity became evident in zymograms of the media after 6 h to 8 h incubation with AA but was already discernible after 4 h incubation with PMA. We then pre-exposed the cells to 10 μM AA for different periods of time after which the medium was discarded. The incubation was then carried on with culture medium without AA for 24 h for MMP-9 activity to be detectable in zymograms. As shown in Fig. 2B, MMP-9 activity was augmented already after 1 h incubation with AA and increased for at least 6 h.

**Figure 3:**
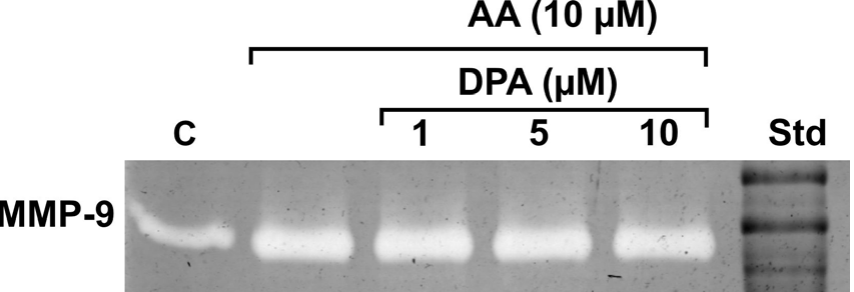
**Effect of docosapentaenoic acid (DPA) on arachidonic acid (AA) stimulated production of MMP-9**. MonoMac 6 cells (0.8 × 10^6 ^cells/ml in X-Vivo 15) were incubated in the presence of 5 μM arachidonic acid and increasing concentrations of DPA (1, 5, 10 μM) for 24 h. The presence of MMP-9 activity in the media was determined using zymography.

Since EPA, DPA or DHA by themselves had no effect on MMP-9 secretion, we looked at their effect on AA-induced production of MMP-9. Co-incubation of 5 μM AA with increasing concentrations of EPA, DPA or DHA for 24 h did not diminish or increase the secretion of MMP-9 (Fig. 3).

**Figure 4:**
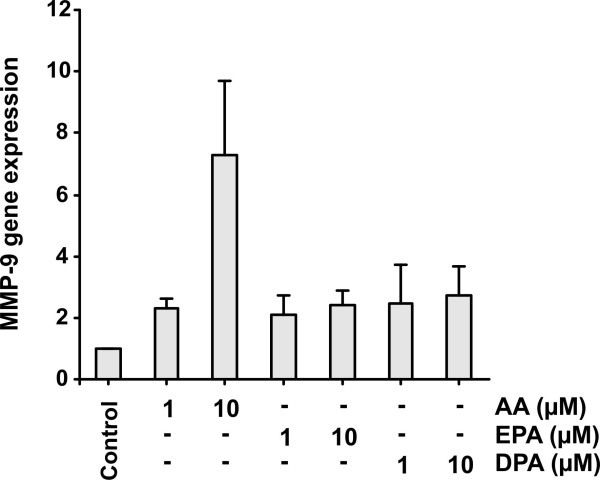
**Effect of polyunsaturated fatty acids on MMP-9 mRNA expression**. MonoMac 6 cells (4 × 10^6 ^cells/5 ml X-Vivo 15 on Nunclon 60 mm dishes) were treated with 1 or 10 μM arachidonic acid (AA), eicosapentaenoic acid (EPA) or docosapentaenoic acid (DPA) for 24 h. Then the cells were pelleted by centrifugation (400 × G, +4°C) and washed with ice-cold PBS. Total cellular RNA was isolated, reverse transcribed and the resulting cDNA was amplified as described in the methods. The MMP-9 expression was demonstrated by real-time PCR. Results represent the means ± SEM of four independent experiments. Only 10 μM AA increased the expression of MMP-9 mRNA statistically significantly.

We then tested the hypothesis that AA could increase the secretion of MMP-9 by modulating its gene expression. In Fig 4 we show that MMP-9 mRNA expression was increased 6- to 8-fold when stimulated with 10 μM AA for 24 h; it was only slightly up-regulated in the presence of n-3 fatty acids and 1 μM AA (Fig. 4). Predictably, PMA treatment stimulated MMP-9 expression to a much greater extent than 10 μM AA (results not shown).

**Figure 5:**
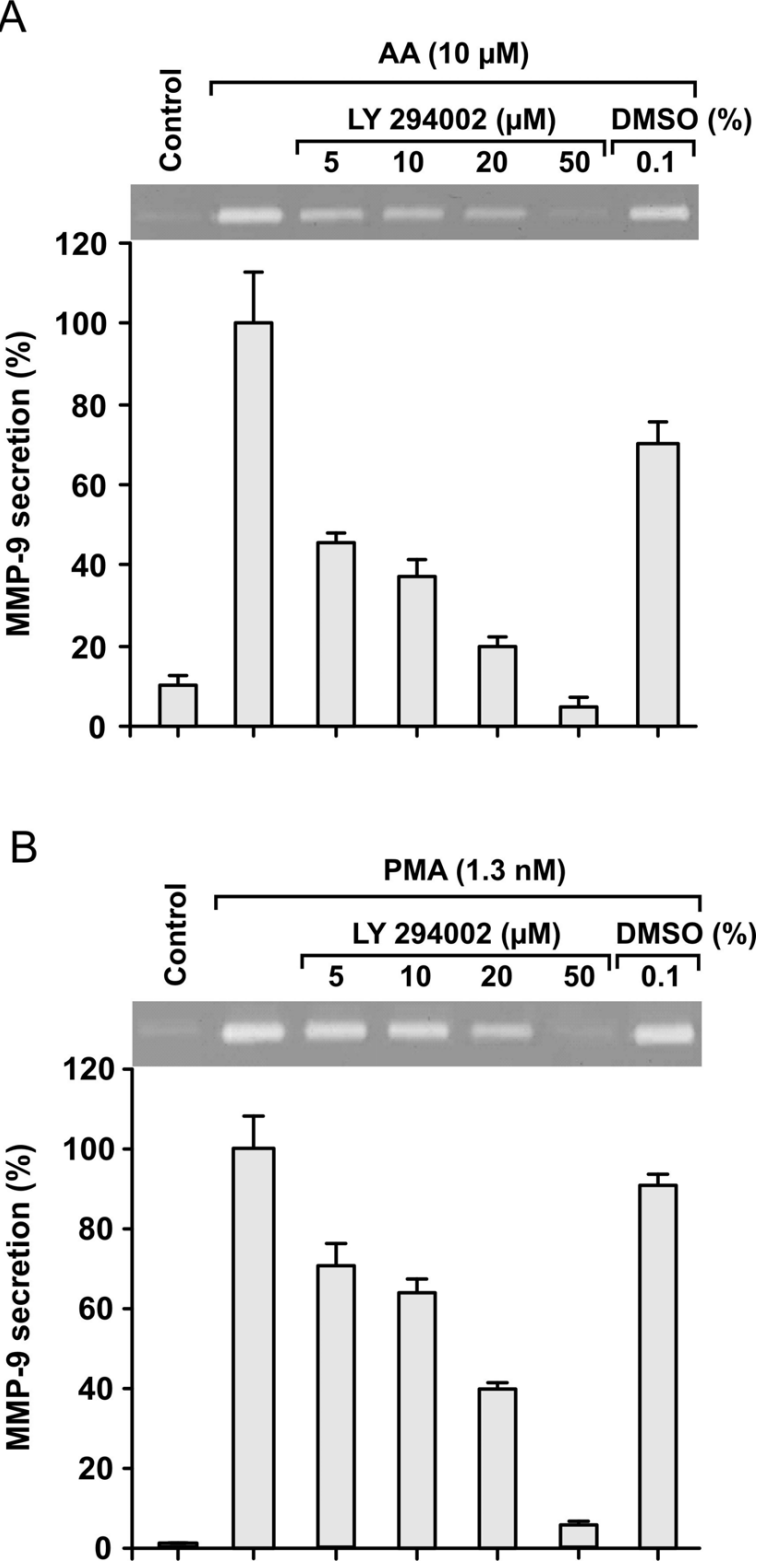
**Inhibition of arachidonic acid (AA) and PMA induced stimulation of MMP-9 secretion by LY294002. Dose- and time-dependent stimulation of MMP-9 secretion by arachidonic acid**. MonoMac 6 cells (0.8 × 10^6 ^cells/ml in X-Vivo 15) were treated with various concentrations of LY294002 in DMSO for 30 min. Then 10 μM arachidonic acid (5A) or 1.3 nM PMA (5B) was added and the incubation was continued for 24 h. The media were analysed for the presence of MMP-9 using zymography and image analysis. Results (mean ± SEM) for 4 to 5 independent experiments are shown. All concentrations of LY294002 decreased the AA or PMA induced secretion of MMP-9 statistically significantly.

In an endeavour to understand further the mechanism through which arachidonic acid acts, we investigated whether it affected the activity of phosphatidylinositol 3-kinase (PI3K). For this end we incubated MonoMac 6 cells with LY 294002, an inhibitor of PI3K for 30 min before adding 10 μM AA or 1.3 nM PMA for 24 h. As shown in Fig 5, Ly 294002 blocked the induction of MMP-9 by AA and PMA in a dose-dependent manner. However, if the inhibitor was removed from the incubation medium before the addition of AA or PMA the inhibitory effect was lost. These results indicate a role for PI3K activation in the signalling of both AA and PMA to produce MMP-9. To test the phophatidylinositol signalling system downstream of PI3K we treated the MonoMac 6 cells for 30 min with various concentrations of phosphatidyl inositol analog, SH-5, an inhibitor of Akt. This was followed by stimulation of the cells with 10 μM AA for 24 h. A concentration dependent decrease of AA-induced MMP-9 secretion was seen in zymographic analysis. The decrease was 20% (± 4%, n = 4) for 2.5 μM, 35% (± 3%, n = 4) for 5 μM and 53% (± 2%, n = 4) for 10 μM SH-5. These results further point to phosphatidylinositol lipids as mediators of AA stimulation.

## Discussion

In the present study we show that incubation of human leukaemia Mono Mac 6 cells in the presence of AA (20:4n-6) in serum-free medium is associated with increased secretion of MMP-9 to the growth medium in comparison with control cells incubated without added AA. The response was concentration-dependent and intensified considerably as the level of AA rises from 1- to 40-μM. Since the composition and content of cellular fatty acids changed in accordance with fatty acids added to the incubation medium, the fatty acids obviously entered the cells either by diffusion or by membrane transport proteins. It is possible that the effect of AA on MMP-9 secretion was mediated via binding to a membrane receptor, since a family of G-protein coupled free fatty acid receptors has been recently identified [16]. However, none of these receptors has been shown to be specific to arachidonic acid.

EPA, DPA or DHA, fatty acids of the n-3 series, which are considered to be anti-inflammatory, had no effect on basal secretion of MMP-9. Since the n-3 series of polyunsaturated fatty acids are known to compete for the same enzymes as the n-6 series fatty acids in a multitude of cellular processes, and the enzymes seem to prefer n-3 series, we tested whether the effect of arachidonic acid on secretion of MMP-9 could be controlled or even prevented by increasing concentrations of EPA, DPA or DHA. However, the AA-induced activity of MMP-9 did not diminish in the presence of n-3 fatty acids, although the cellular fatty acid content revealed an ample availability of n-3 fatty acids after incubation. It is possible, though, that the intracellular pathways of n-6 and n-3 fatty acids differ in ways that give advantage to AA [17,18]. On the other hand, it is also possible that a prolonged exposure of cells to n-3 fatty acids is necessary for inhibition of AA-triggered MMP-9 activity [19].

Experiments where MonoMac 6 cells were pre exposed to AA for increasing periods of time (Fig. 2B) showed that the system delivering MMP-9 to the medium was activated already after 1 h incubation and increased for at least 6 h. Given that a quantity of pro-MMP-9 is stored in intracellular vesicles it is possible that the activity detected during the first hour of incubation was in fact due to degranulation of existing vesicles. Degranulation of storage vesicles has been shown to occur quickly in response to less than 1 h treatment with IL-8 in neutrophils, where the activity of MMP-9 increased 2.5-fold but then declined [20]. It is not known whether AA causes degranulation in MonoMac 6 cells, but the continued accumulation of MMP-9 in the incubation medium speaks for additional mechanisms. The increases in AA-induced secretion of MMP-9 was blocked by co-treatment of MonoMac 6 cells with 1–10 μM of the protein synthesis inhibitor cycloheximide (data not shown), indicating further that the AA effect requires *de novo *protein synthesis.

The transcriptional regulatory mechanism was confirmed by the increase of MMP-9 mRNA by AA treatment. We could show in repeated experiments that the expression level of MMP-9 mRNA increased in response to AA, whereas EPA or DPA did not produce such amplifications. The response with AA was somewhat variable as can be seen from Fig. 4, but it was well associated with the results of enzyme activity as measured using zymography. It is notable that the level of mRNA was persistently elevated after 24-h incubation in the presence of the AA in comparison with basal conditions.

Production of MMP-9 by macrophages has been shown to occur through a prostaglandin E_2_/cAMP-dependent mechanism. It has been shown that production of MMP-9 is induced by prostaglandin E_2 _(PGE_2_), the predominant eicosanoid of macrophages [21]. PGE_2 _is produced from AA by the coupled actions of cyclooxygenase and prostaglandin E synthase [22]. Cylooxygenase 1 (COX-1) is constitutively expressed but cyclooxygenase 2 (COX-2) is induced by inflammatory cytokines, growth factors or phorbol esters. PGE_2 _acts in an autocrine or paracrine manner and activates adenylate cyclase. The increased cAMP leads to activation of PKA, and the expression of MMP-9 [23]. Studies using human monocytes have shown that induction of COX-2 protein production and concomitant PGE_2 _increase occur at 4 h – 16 h after stimulation with LPS [24]. In contrast, in our experiments the secretion of MMP-9 was enhanced after 1 h incubation in AA containing medium, speaking for a faster mechanism. Also, it has been shown that PGE_2 _alone does not stimulate MMP-9 synthesis in MonoMac 6 cells but needs a simultaneous presence of an effective primary stimulus such as TNFα [25].

The n-3 series fatty acids are competitive inhibitors of cyclooxygenase-catalysed catabolism of AA and show a preference for the COX-2 over the COX-1 forms [26], but could not prevent the effect of AA in our experiments. Therefore, we felt that AA by itself could trigger the events that lead to MMP-9 secretion. Earlier studies by Hii et al. [27] have shown that AA or its metabolites can activate PI3K-mediated pathways. On the other hand, studies of Lu et al. [28] show that in LPS-activated human peripheral blood monocytes MMP-9 production resulted through the stimulation of PI3K signalling pathway. Indeed, when we blocked the capability of PI3K to generate PIP3 in MonoMac 6 cells by Ly 294002 (a specific inhibitor of PI3K), MMP-9 production was inhibited in both AA and PMA stimulated cells. The result indicates that the PI3K pathway has a central role in regulation of monocyte MMP-9 production following stimulation with AA. This outcome accords with the results of Sato and co-workers, where the secretion of MMP-9 could be prevented by nobiletin (a flavonoid) or LY294002 in fibrosarcoma cells [29]. Activation of PI3K leads to phosphorylation of inositol lipids, in response to activation and membrane translocation of Akt/Protein kinase B. Inhibition of Akt by SH-5 was associated with attenuation of MMP-9 secretion in AA stimulated MonoMac 6 cells. This result further points to the involvement of PI3K signalling system in AA-induced MMP-9 production. Activated Akt in turn phosphorylates several proteins that regulate cell survival, including Bad and caspase 9 [30]. This activation is important for monocyte survival and therefore the results using PI3K or Akt inhibitors should be considered very carefully. It is possible that MonoMac 6 cell viability was affected and therefore the cells stopped secreting MMP-9. However, no evidence of increased cell death could be seen in Trypan Blue exclusion tests. MMP-9 production in response to LPS-induced PI3K stimulation has also been reported to occur through IKKα/NFκB pathway in human monocytes [28]. Whether AA also acts through this pathway remains to be examined.

AA concentrations in tissues and for example in inflammatory cells are dictated in part by the concentration of AA that these cells are exposed to and partly by local formation from linoleic acid [31]. AA is distributed to the cells as albumin-bound FA or lysophospholipid and as a component of various lipoprotein particles. Extracellular and intracellular concentrations of free AA are kept low, but in stressful situations like inflammation the extracellular concentration of AA can reach up to a level of a hundred μM [32]. This means that the fatty acid concentrations that were used in our in vitro incubations were well within the in vivo range. The primary pathway leading to AA release is the hydrolysis of phospholipids by enhanced activity of either secretory or cytoplasmic phospholipase A2. Relevant to the study of atherogenesis is the finding in human THP-1 monocytes that native LDL elicited a rapid, dose-dependent release of AA which was further increased when LDL was modified by secretory phospholipase A2 (PLA2) [33]. Also, it was recently reported by Namgaladze et al. [34] that sPLA2-modified LDL activated the PI3K pathway, thus increasing monocyte survival. The active ingredient was shown to reside in the lipid fraction and was suggested to be AA since this fatty acid reproduced the effect. Our results further establish the important role of AA in intracellular communications system and as a pro-atherogenic factor by linking it to the production of matrix degrading protease MMP-9 by way of PI3K.

## Conclusion

In summary, MonoMac 6 cells secreted MMP-9 in response to increasing clinically relevant concentrations of AA in the medium. The secretion increased due to increased expression of MMP-9 mRNA. MMP-9 secretion started fairly rapidly and could not be prevented by simultaneous presence of n-3 series fatty acids. The secretion could be attenuated by LY 298002, a specific phosphatidylinositol-3-kinase inhibitor and by SH-5, a selective Akt inhibitor, which suggests that activation of phosphatidylinositol-3-kinase by AA leads to augmented and sustained MMP-9 production.

## Methods

### Cell culture

MonoMac 6, an established human monocytic cell line that grows in suspension, was obtained from the German Collection of Microorganisms and Cell Cultures (DSMZ ACC 124; Braunschweig, Germany). Cell cultures were maintained in RPMI-1640 medium (BioWhittaker, Belgium) supplemented with 2 mM L-glutamine, 5 ml/l non-essential amino acids, penicillin (50 IU/ml), streptomycin (100 μg/ml), 1 mM sodium pyruvate, 1 mM oxaloacetate, 0.2 U/ml bovine insulin (OPI Media Supplement, Sigma Chemicals Co) and 10% heat-inactivated fetal bovine serum (FBS). The cells were cultured at a density of 0.3 – 1 × 10^6 ^cells/ml in humidified 5% CO_2 _at 37°C. Fresh medium was added to the cultures twice weekly replacing one third of the suspension which was either reseeded or discarded. For the experiments, cells were harvested, washed with phosphate buffered saline (PBS), pH 7.4, and seeded on 12-well Nunclon plates (Nunc A/S, Denmark) at 0.8 × 10^6 ^cells/ml in serum-free medium X-Vivo 15 (BioWhittaker, Belgium) supplemented with penicillin-streptomycin. The cells were incubated in the presence of indicated concentrations of fatty acids (AA, EPA, DPA, DHA, all from Cayman Chemical), PMA (Sigma), LY 294002 (Cayman Chemical) or SH-5 (Alexis Biochemicals) for 24 h unless otherwise stated. Cell viability was checked with the Trypan Blue exclusion test. Cellular protein concentrations were determined by the method of Lowry.

### Zymography

Gelatin zymography was essentially done as described [35]. Media from MonoMac 6 incubations were diluted in electrophoresis sample buffer [36] and subjected to electrophoresis in a 10% SDS-polyacrylamide gel embedded with 1 mg/ml gelatin (Sigma) in nonreducing conditions. We used the Mini-PROTEAN II apparatus at 4°C (Bio Rad). After electrophoresis the enzymes were renatured with two 30 min washes in 0.25% Triton X-100, and then the enzyme reaction was allowed to proceed in activation buffer (50 mM Tris-HCl, pH 7.5, containing 15 mM CaCl_2_, 1 μM ZnCl_2 _and 1% Triton X-100) at 37°C for 18 h. Thereafter the gels were stained for 1.5 h with 0.1% (w/v) Coomassie Brilliant Blue in 40% (v/v) isopropanol and destained in 7% acetic acid for a minimum of 3 h. As a molecular weight marker we used BenchMark™ Protein Ladder from Invitrogen.

### Image analysis

The degree of gelatin digestion was quantified using an Epson Perfection 3200 Photo scanner interfaced to a computer. Gels were scanned using Epson Scan software (version 1.00E) in transparency option and gray scale mode. The density of each pixel is encoded on a scale ranging from 1 (clear) to 255 (opaque). The scanner was calibrated using the Stouffer Graphic Arts step tablet. The images were analysed with Scion Image software (Scion Corporation). The density measurements were linearized using the Rodbard curve-fitting function within the Scion Image program. The images were digitally inverted so that integration of bands is reported as positive values. The pixel density was determined after background density subtraction and used to calculate the integrated density of a selected band. The integrated density values of gelatinolytic activity of MMP-9 are reported in volume units of pixel intensity per mm^2 ^and adjusted for cellular protein concentration.

### RNA

Total RNA was isolated from fresh cells using a commercial kit (Invitrogen Ltd, Paisley, UK). After isolation, RNA was incubated with DNAse I to remove genomic DNA. To determine the concentration and purity of RNA, the absorbance was measured at 260 nm and 280 nm in a spectrophotometer from an aliquot of the samples. Before cDNA synthesis, the integrity and size distribution of total RNA was checked by agarose gel electrophoresis and ethidium bromide staining.

### cDNA synthesis and RT-PCR

Total RNA was reverse transcribed to cDNA using TagMan reverse transcription reagents (Roche Molecular Systems, Inc, Branchburg, NJ, USA).

Relative quantification of MMP-9 mRNA levels was done using real time PCR (AbiPrism 7000, Applied Biosystems, Foster City, CA, USA) with TagMan Assays-on-demand probe process (assay ID Hs00234579-m1). Eukaryotic translation elongation factor (EEF-2) mRNA was used as an endogenous RNA control (assay ID Hs00157330-m1). Relative quantification of the target MMP-9 mRNA levels in comparison to the reference gene EEF-2 (ratio) was calculated using the comparative C_T _method. The results were expressed as N-fold differences in MMP-9 expression relative to EEF-2 expression.

### Analysis of cellular fatty acid composition

All organic solvents were redistilled. Chloroform, methanol, toluene (all analytic grade) were purchased from Merck (Darmstadt, Germany). Petroleum spirit (b.p. 60–69°C) was from Neste Oy (Finland).

The cells were stored under nitrogen at -70°C until analysis. Lipids were extracted from approximately 1.2 × 10^6 ^cells with chloroform-methanol, partitioned, and the chloroform phase was dried under N_2 _[37]. Tripentadecanoic acid was added as an internal standard. Saponification of lipids was carried out by boiling in 33% KOH in ethanol at 85°C for 2 h. The nonsaponifiable lipids were extracted into petroleum spirit. The lower phase was acidified with HCl, and the fatty acids were extracted into petroleum spirit. After evaporation of the solvent in a stream of nitrogen, the residue was dissolved in toluene and the fatty acids were esterified with 2% H_2_SO_4_-methanol at 80°C for 2 h. The methyl esters of fatty acids were extracted into petroleum spirit. The fatty acids were analysed with Hewlett-Packard 5890A gas chromatograph equipped with a flame ionisation detector using a Nordion NB 351 capillary column (25 m, 0.32 mm I.D., 0.20 μm).

### Data analysis

Results are expressed as mean ± SEM. Statistical comparisons were made by one-way analysis of variance using SPSS software, version 16.0. Post hoc comparisons of means were made using Dunnett's test. A p value < 0.05 was taken to be statistically significant.

## Competing interests

The authors declare that they have no competing interests.

## Authors' contributions

TS and OJ had substantial contributions to conception and design and interpretation of data and writing the manuscript. TK and STN had substantial contributions to conception and design. TS, TK and SK carried out the biochemical analyses. All authors read and approved the final manuscript.

## References

[B1] Massaro M, Scoditti E, Carluccio MA, De Caterina R (2008). Basic mechanisms behind the effects of n-3 fatty acids on cardiovascular disease. Prostaglandins Leukot Essent Fatty Acids.

[B2] De Caterina R, Madonna R, Bertolotto A, Schmidt EB (2007). N-3 Fatty Acids in the Treatment of Diabetic Patients: Biological Rationale and Clinical Data. Diabetes Care.

[B3] Calder PC (2008). Polyunsaturated fatty acids, inflammatory processes and inflammatory bowel diseases. Mol Nutr Food Res.

[B4] Serhan CN, Yacoubian S, Yang R (2008). Anti-inflammatory and proresolving lipid mediators. Annu Rev Pathol.

[B5] Wal AC van der, Becker AE (1999). Atherosclerotic plaque rupture – pathologic basis of plaque stability and instability. Cardiovasc Res.

[B6] Galis ZS, Sukhova GK, Lark MW, Libby P (1994). Increased expression of matrix metalloproteinases and matrix degrading activity in vulnerable regions of human atherosclerotic plaques. J Clin Invest.

[B7] Anonymous (1999). Dietary supplementation with n-3 polyunsaturated fatty acids and vitamin E after myocardial infarction: results of the GISSI-Prevenzione trial. Gruppo Italiano per lo Studio della Sopravvivenza nell'Infarto miocardico. Lancet.

[B8] Hooper L, Thompson RL, Harrison RA, Summerbell CD, Ness AR, Moore HJ, Worthington HV, Durrington PN, Higgins JP, Capps NE, Riemersma RA, Ebrahim SB, Davey Smith G (2006). Risks and benefits of omega 3 fats for mortality, cardiovascular disease, and cancer: systematic review. BMJ.

[B9] Deckelbaum RJ, Worgall TS, Seo T (2006). N-3 Fatty Acids and Gene Expression. Am J Clin Nutr.

[B10] Thies F, Garry JM, Yaqoob P, Rerkasem K, Williams J, Shearman CP, Gallagher PJ, Calder PC, Grimble RF (2003). Association of n-3 polyunsaturated fatty acids with stability of atherosclerotic plaques: a randomised controlled trial. Lancet.

[B11] Clohisy JC, Connolly TJ, Bergman KD, Quinn CO, Partridge NC (1994). Prostanoid-induced expression of matrix metalloproteinase-1 messenger ribonucleic acid in rat osteosarcoma cells. Endocrinology.

[B12] Medina L, Perez-Ramos J, Ramirez R, Selman M, Pardo A (1994). Leukotriene C4 upregulates collagenase expression and synthesis in human lung fibroblasts. Biochim Biophys Acta.

[B13] Rajah R, Nunn SE, Herrick DJ, Grunstein MM, Cohen P (1996). Leukotriene D4 induces MMP-1, which functions as an IGFBP protease in human airway smooth muscle cells. Am J Physiol.

[B14] Vaday GG, Hershkoviz R, Rahat MA, Lahat N, Cahalon L, Lider O (2000). Fibronectin-bound TNF-alpha stimulates monocyte matrix metalloproteinase-9 expression and regulates chemotaxis. J Leukoc Biol.

[B15] Huhtala P, Tuuttila A, Chow LT, Lohi J, Keski-Oja J, Tryggvason K (1991). Complete structure of the human gene for 92-kDa type IV collagenase. Divergent regulation of expression for the 92- and 72-kilodalton enzyme genes in HT-1080 cells. J Biol Chem.

[B16] Briscoe CP, Tadayyon M, Andrews JL, Benson WG, Chambers JK, Eilert MM, Ellis C, Elshourbagy NA, Goetz AS, Minnick DT, Murdock PR, Sauls HR, Shabon U, Spinage LD, Strum JC, Szekeres PG, Tan KB, Way JM, Ignar DM, Wilson S, Muir AI (2003). The orphan G protein-coupled receptor GPR40 is activated by medium and long chain fatty acids. J Biol Chem.

[B17] Moghaddami N, Irvine J, Gao X, Grover PK, Costabile M, Hii CS, Ferrante A (2007). Novel action of n-3 polyunsaturated fatty acids: inhibition of arachidonic acid-induced increase in tumor necrosis factor receptor expression on neutrophils and a role for proteases. Arthritis Rheum.

[B18] Cao Y, Traer E, Zimmerman GA, McIntyre TM, Prescott SM (1998). Cloning, expression, and chromosomal localization of human long-chain fatty acid-CoA ligase 4 (FACL4). Genomics.

[B19] Massaro M, Habib A, Lubrano L, Del Turco S, Lazzerini G, Bourcier T, Weksler BB, De Caterina R (2006). The omega-3 fatty acid docosahexaenoate attenuates endothelial cyclooxygenase-2 induction through both NADP(H) oxidase and PKC epsilon inhibition. Proc Natl Acad Sci USA.

[B20] Opdenakker G, Steen PE Van den, Dubois B, Nelissen I, Van Coillie E, Masure S, Proost P, Van Damme J (2001). Gelatinase B functions as regulator and effector in leukocyte biology. J Leukoc Biol.

[B21] Corcoran ML, Stetler-Stevenson WG, DeWitt DL, Wahl LM (1994). Effect of cholera toxin and pertussis toxin on prostaglandin H synthase-2, prostaglandin E2, and matrix metalloproteinase production by human monocytes. Arch Biochem Biophys.

[B22] Cipollone F, Prontera C, Pini B, Marini M, Fazia M, De Cesare D, Iezzi A, Ucchino S, Boccoli G, Saba V, Chiarelli F, Cuccurullo F, Mezzetti A (2001). Overexpression of functionally coupled cyclooxygenase-2 and prostaglandin E synthase in symptomatic atherosclerotic plaques as a basis of prostaglandin E(2)-dependent plaque instability. Circulation.

[B23] Saja K, Chatterjee U, Chatterjee BP, Sudhakaran PR (2007). Activation dependent expression of MMPs in peripheral blood mononuclear cells involves protein kinase A. Mol Cell Biochem.

[B24] Demasi M, Caughey GE, James MJ, Cleland LG (2000). Assay of cyclooxygenase-1 and 2 in human monocytes. Inflamm Res.

[B25] Vaday GG, Schor H, Rahat MA, Lahat N, Lider O (2001). Transforming growth factor-beta suppresses tumor necrosis factor alpha-induced matrix metalloproteinase-9 expression in monocytes. J Leukoc Biol.

[B26] Ringbom T, Huss U, Stenholm A, Flock S, Skattebol L, Perera P, Bohlin L (2001). Cox-2 inhibitory effects of naturally occurring and modified fatty acids. J Nat Prod.

[B27] Hii CS, Moghadammi N, Dunbar A, Ferrante A (2001). Activation of the phosphatidylinositol 3-kinase-Akt/protein kinase B signaling pathway in arachidonic acid-stimulated human myeloid and endothelial cells: involvement of the ErbB receptor family. J Biol Chem.

[B28] Lu Y, Wahl LM (2005). Production of matrix metalloproteinase-9 by activated human monocytes involves a phosphatidylinositol-3 kinase/Akt/IKKalpha/NF-kappaB pathway. J Leukoc Biol.

[B29] Sato T, Koike L, Miyata Y, Hirata M, Mimaki Y, Sashida Y, Yano M, Ito A (2002). Inhibition of activator protein-1 binding activity and phosphatidylinositol 3-kinase pathway by nobiletin, a polymethoxy flavonoid, results in augmentation of tissue inhibitor of metalloproteinases-1 production and suppression of production of matrix metalloproteinases-1 and -9 in human fibrosarcoma HT-1080 cells. Cancer Res.

[B30] Song G, Ouyang G, Bao S (2005). The activation of Akt/PKB signaling pathway and cell survival. J Cell Mol Med.

[B31] Zhou L, Nilsson A (2001). Sources of eicosanoid precursor fatty acid pools in tissues. J Lipid Res.

[B32] Brash AR (2001). Arachidonic acid as a bioactive molecule. J Clin Invest.

[B33] Oestvang J, Bonnefont-Rousselot D, Ninio E, Hakala JK, Johansen B, Anthonsen MW (2004). Modification of LDL with human secretory phospholipase A(2) or sphingomyelinase promotes its arachidonic acid-releasing propensity. J Lipid Res.

[B34] Namgaladze D, Brune B (2006). Phospholipase A2-modified low-density lipoprotein activates the phosphatidylinositol 3-kinase-Akt pathway and increases cell survival in monocytic cells. Arterioscler Thromb Vasc Biol.

[B35] Kenagy RD, Nikkari ST, Welgus HG, Clowes AW (1994). Heparin inhibits the induction of three matrix metalloproteinases (stromelysin, 92-kD gelatinase, and collagenase) in primate arterial smooth muscle cells. J Clin Invest.

[B36] Laemmli UK (1970). Cleavage of structural proteins during the assembly of the head of bacteriophage T4. Nature.

[B37] Folch J, Lees M, Sloane Stanley GH (1957). A simple method for the isolation and purification of total lipides from animal tissues. J Biol Chem.

